# Volunteerism during humanitarian crises: a practical guide

**DOI:** 10.1186/s13054-022-03984-4

**Published:** 2022-04-19

**Authors:** Heatherlee Bailey, Lewis J. Kaplan

**Affiliations:** 1 Department of Emergency Medicine, Durham VA Medical Center, 508 Fulton St., Durham, NC 27705 USA; 2 Division of Trauma, Surgical Critical Care and Emergency Surgery, Perelman School of Medicine, University of Pennsylvania, Philadelphia, PA USA; 3 Surgical Services, Division of Surgical Critical Care, Corporal Michael J Crescenz VA Medical Center, Philadelphia, PA USA

**Keywords:** Volunteerism, Humanitarian, Disaster, Safety, Crisis, Conflict

## Abstract

Volunteerism to provide humanitarian aid occurs in response to disasters, crises, and conflict. Each of those volunteerism triggers engenders personal risk borne by the healthcare volunteer while rendering aid and merit specific evaluation. Factors that impact decision-making with regard to volunteering are personal, structural and crisis specific. Practical approaches to travel and on-scene safety benefit volunteers and should inform planning and preparation for volunteerism-driven travel. These approaches include planning for evacuation and potential rescue. These unique skills and approaches are generally not part of medical education outside of military service. The global medical community, including medical professional organizations, should embrace this opportunity to improve medical education and professional development to support humanitarian aid volunteerism. Disaster, crisis, or conflict-driven healthcare volunteerism highlights the core elements of altruism, dedication, and humanity that permeate clinician’s drive to render aid and save lives.

Disasters and crises, including military conflict, often trigger the healthcare professional’s inherent desire to render aid and save lives [1]. While many donate money and supplies others wish to render care for victims. Local clinicians understand the realities of care near where they practice. Others will need to travel from nearby countries, or a different continent. Volunteers help in three major locales: (1) unimpacted facilities overwhelmed by victims or refugees, (2) temporary shelters or care camps, and (3) conflict zones (more rare). The recent SARS-CoV-2 pandemic demonstrated altruism in health care as volunteers helped deliver care around the world—even ahead of evidence-based therapeutics. [2] This space, the established acute care facility—is comfortable for clinicians as it reflects their daily practice. Crisis volunteers, especially when there is armed conflict, are unlikely to solely render care within the confines of a hospital. Indeed, care outside of a hospital is quite distinct from most clinician’s usual practice and merits specific preparation to support safety while serving as an asset—and not a liability—during relief efforts. Unfortunately, this aspect of professional practice is rarely addressed during graduate medical education or subsequent training in most disciplines. Given the current landscape of health care needs, providing volunteer-relevant education and training represents an opportunity the global medical community should embrace as lives are at risk.

The most important decision a potential volunteer must make is whether to travel to a crisis location (Fig. 1). One must assess safety threats, potential mitigation strategies, personal risk tolerance, as well as the specific crisis type. [3] Some crises are more time limited (natural disaster) compared with others (pandemic), while military conflicts present a vastly accelerated risk profile. Major personal risks to be considered include being in a location remote from family or usual resources, illness, injury, disability, or death. Thus, a personal risk tolerance assessment is an ideal point of embarkation prior to volunteering. The specific location may influence decision-making with regard to safety as well. Geographically adjacent but unimpacted hospitals or refugee camps are less risky than conflict zones. Most volunteers link with a group such as a non-governmental organization, hospital’s team, medical professional organization’s volunteer group; military or police threat mitigation while volunteering is variable. It is uncommon and generally inadvisable to travel solo as a volunteer, especially if one intends to engage in health care. Governmental approval to provide basic or complex care is required and mitigates against medical liability.

**Fig. 1:**
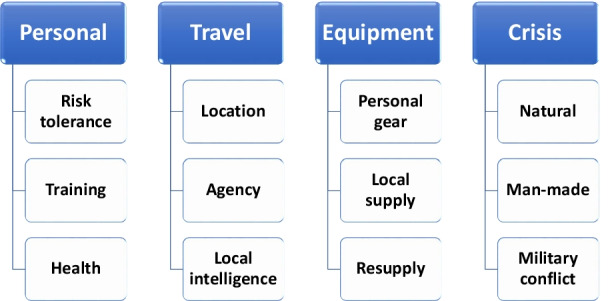
Key factors influencing the decision to volunteer during a crisis. This graphic depicts key considerations that may influence a clinician's decision-making regarding volunteering during a crisis. Major domains include those unique to the clinician (personal), travel considerations, as well as equipment issues. Natural and man-made disasters commonly entail less personal risk than militarily-driven crises

Other elements that should inform one’s decision-making include, but are not limited to: prior volunteering experience, specific training for out-of-hospital aid or rescue activity, local language fluency, anticipated team members, communication infrastructure, supply and equipment availability, environmental conditions including temperature and terrain, local intelligence reports and data, personal health and fitness, personal gear or lack thereof, and availability to be absent from one’s usual site of employment [4, 5]. Lack of experience or training may strongly drive post-traumatic stress disorder in surviving volunteers unprepared for the sensory impact of the realities of disaster or conflict aid [6, 7]. In particular, injuries may be quite gruesome, and the inability to rescue everyone—especially children—exacts a vast emotional toll that may be dehumanizing. Local intelligence may flow from official government or traveler sites, especially if the location was a prior vacation destination. Due to the pandemic, some travel sites where individuals can log helpful contacts, professional services, danger spots, as well as a chronicle of their experiences have been shuttered [8]. Terrain and weather conditions remain available via a number of web-based services. The absence of personal equipment should give one pause, and a rapid acquisition of gear with which one is not well acquainted may be quite dangerous. Moreover, conflict zones place volunteers in a vulnerable position; care facilities, even impromptu ones, may be purposefully or inadvertently targeted by military action or violent extremism [9, 10]. The occurrence of such acts spurred the United Nations Security Council 2016 resolution that addressed the protection of civilians -including humanitarian aid volunteers—during armed conflict. Once a clinician has decided to volunteer, vast preparation is required.

Preparation should address gear, documents, communication, electrical power, medical travel insurance, personal medications, travel to the site(s), and planning for evacuation or rescue; Table 1 provides a checklist of items explored below. Bring gear that is durable, waterproof, easily portable, and is designed to not attract attention (avoid designer labels or unique designs) to help avoid being a victim of interpersonal violence. Backpacks optimally provide rear entry and slash-resistance to deter pickpockets. Single shoulder wear of a backpack is much more safe than the more ergonomically correct and comfortable two shoulder carry. Backpacks are common targets for thieves who may grab and pull the pack off of you. A single shoulder carry allows the pack to slide off instead of pulling you to the ground, or into an immobile object. Pants and vests in particular should have a variety of pockets, some of which should zip closed to help retain key items. Waterproof boots with a steel or otherwise non-crushable toe and a puncture-resistant and nonskid sole are essential. Depending on climate and locale, an appropriate packable hat, gloves, sunglasses as well as durable non-fogging safety goggles should be included in your gear list. Avoiding bringing jewelry other than an inexpensive unadorned wedding band or an inexpensive analog watch decreases theft potential as well as personal injury risk.

**Table 1:** Checklist for packing for volunteering during a humanitarian crisis

Category	Item
Documents	Driver’s license
	Medical license
	Health insurance
	Medical travel insurance
	Passport including vaccination status documentation (COVID, yellow fever, etc.)
	VISA (if needed)
	Itinerary and local contact(s)
	Emergency contact(s) list
	Plane or boat tickets
Equipment	Backpack
	Money belt (or similar)
	Headlamp
	Tourniquet
	Vehicle window breaker/seat belt cutter
	Smartphone (new)
	Solar charging or hand-crank device
	Rainsuit ± insulation (depending on locale)
	Thermal blanket (depending on locale)
	Water purification device
	Hat, gloves, sunglasses, goggles (depending on locale)
	Pants with multiple pockets (some should zip closed)
	Vest with multiple pockets (some should zip closed)
	Boots (waterproof, non-crushable toe, puncture resistant, nonskid sole)
	Personal medications (3X’s the amount required for length of deployment)
	Device to secure door locking mechanism (if staying in a hotel)
	Local map with 2 routes of egress clearly marked
	Emergency locator beacon labelled using the local language
	Satellite phone or sat phone cradle for a smartphone
	Small denomination local currency including a “give-away” roll
	RFID wallet or sleeve for chip-based credit card or license
	Internet access device
	Prepackaged food (i.e., protein bars, etc.)
	Inexpensive watch
	Plain jewelry (if any)

Pack a tourniquet, headlamp, money belt (or similar device), and personal safety devices like a window breaker/seat belt cutter as part of your routine gear. These devices allow you to be prepared to render aid, navigate when there is no power (and still use both hands), and escape entrapment in a vehicle that has crashed. Spread out money (small denominations preferred) across different locations on your body and have a “give-away” roll of small denominations in a larger roll in an accessible pocket if confronted; your personal safety is of utmost importance. Paper documents should travel with you but should be also stored on a smartphone, and accessible via a cloud-based server. Such documents include: driver’s license, medical license, passport, visa (if required), health insurance, medical travel insurance, emergency contact(s), itinerary, and local contact(s). An RFID-blocking sheath or wallet protects chip-based cards. Secure a new phone devoid of personal information for volunteering to help derail identity theft.

Communication preparation includes both devices and strategies [11] Cell towers may not be operational, rendering the need for a satellite phone, or a smartphone satellite adapter. Such devices are available for rent instead of purchase. A satellite-linked emergency beacon should remain tethered to you and should be labeled with simple directions using the local language (if you are found unconscious and cannot press the signal button to trigger rescue). Solar collection panels to charge batteries, or a hand-held rotary device to generate power for electronics, are essential as the local power grid may be unstable or entirely absent [12]. A communication plan is not generally part of daily life and requires a change in perspective that reflects the unique risks associated with a non-native locale during a period of instability. Notify home emergency contacts at the start and end of every travel segment (at the airport, departing, landed at destination, cleared customs, etc.). This provides a sequential log of where you were when you were last in contact if contact is no longer achievable. Similarly, notify on-site team members whenever leaving from or returning to a location including the anticipated time it will take to finish the journey (leaving to retrieve laundry, 10 min); WhatsApp is encrypted and works everywhere but China (WeChat is instead required). Failure to check-in at the anticipated time should prompt emergency inquiry and rescue if needed. While solo travel is the norm at home, while volunteering one should never travel alone. A “buddy system” approach increases safety from untoward events such as robbery and kidnapping.

Phone safety is often underappreciated as smartphone use characterizes daily life. Avoid using a phone while walking or riding in a vehicle as it will impede situational awareness. Similarly, earbuds of any variety further degrade one’s ability to monitor the surrounding environment and are to be avoided. Phone use while walking should only occur with one’s back firmly placed against a building so that the other three sectors (270°) remain visible. Do not walk with a phone clasped in the hand; replace it in you gear so that it is not visible. Obvious displays wealth may invite interpersonal conflict and potential injury.

Injuries or illness may occur while volunteering, and one must be prepared to manage them both locally as well as with medical evacuation [13, 14]. One’s home health insurance may not cover illness or injury abroad, reinforcing the need for specific medical travel insurance. Given that there are a variety of companies that offer such insurance, ensure that the selected travel insurance covers both local care and medical transport for repatriation. Many plans only cover local road traffic accidents, the event that leads to the greatest loss of traveler life. Therefore, always wear a seat belt and one should decline to travel in a vehicle devoid of a safety belt; a three-point safety harness is preferred. Recall that Emergency Medical Services to transport injured victims to an acute care facility may be absent or severely disrupted during disasters or crises. With military conflict, the destination facility may have been destroyed or rendered non-functional or inaccessible. If one has chronic health conditions that require medication management, one must bring a sufficient supply to span twice the anticipated volunteer tour as departure may be problematic in the desired time frame. When assessing how much medication to bring, a safe approach is to pack three times the medications you anticipated needing; one-third remains at base, one-third travels with you, and one-third serves as resupply for medication losses or departure delays.

Travel to the volunteer site and back home typically relies on the clinician to arrange. This includes travel from the airport or ship terminal to the volunteer base of operations. Schedule driving services from an established service provider ahead of arrival and avoid using impromptu drivers. Ensure that your supplies travel with you by watching luggage get placed in the vehicle before entering the car or van. Plan a tiered set of actions if the driver is not proceeding to the desired location. This requires the volunteer to identify reasonable routes ahead of arrival. Responses can span distracting conversation, requests for rerouting to view specific landmarks, to vehicle exit (even at moderate speed) to physical intervention as a last resort.

Since sites of humanitarian aid may be unsafe due to weather, local phenomena (additional earthquake or flood), or military conflict, planning for emergency evacuation or rescue is essential. Travel to the volunteer site (if different from the base of operations) with a “bug out” bag that contains medications (if needed), water (or a purification device), communication device(s) and power aids, as well as food for three days [15] Shelter may be more problematic to transport and a waterproof suit and a heat-retaining collapsible blanket may instead suffice. In the event of emergency evacuation, mobility is favored over durable shelter. Predetermine at least two egress routes from your volunteer location and base of operations and chart them on a map stored in the bag; do not rely on an electronic device for directions. Identify trustworthy local contacts (if present) and access numbers in advance, including law enforcement, emergency medical services, and your local embassy (if it is still staffed). Some specialty companies provide full-service extraction options (including hostile threat scenarios) but are generally prohibitively expensive for the volunteer. If such services are a serious concern, volunteering in that location should be strongly reconsidered.

Volunteer groups or organizations generally provide food, water, shelter, and travel to care sites within the local region [16]. Shelter may include a hotel, a repurposed building, a portable emergency shelter, or a tent. Hotels are commonly used to house volunteers in a single location, but present safety concerns often not considered during routine travel. Walk the hotel fire escape to ensure that it is not locked should egress be required. Count the steps required to reach the fire escape stairs from your room since fire (or other loss of power) may impede exit visualization. Prevent room invasion by a method that foils disabling the door emergency lock, or wedges the door closed. Many travelers access the hotel Wi-Fi when they enter their room and log-off upon exit. Avoid using hotel Wi-Fi as it is controlled by the hotel and signals when you are/are not present; instead travel with a stand-alone portable device for internet access. Repurposed buildings may have similar safety concerns, but may not have a door lock. Similarly, tent safety is compromised by both the lack of a door and environment instability including heat, cold, and unless suitably treated or designed, water; waterproof clothing may be life-saving. Non-urban remote locations bring distinct threats from local wildlife, insects, and plants for which the volunteer should plan, and which may be relevant for evacuation while awaiting rescue. Avoid sheltering near a water hole as predators will also use that site for hydration; higher ground away from a well-worn trail is preferred.

On-site supplies such as medications, instruments, and intravenous fluids are provided by the hosting organization (when there is one). Similarly, restocking at care sites as well as supply depot security should be provided by the hosting organization which may be the national government or a more local agency. When there is no organization to coordinate activity, ensure scene safety, or direct resupply the humanitarian aid effort may be crippled. Thus, confirming that there is an overarching agency is paramount for clinician volunteers to provide care commensurate with their capabilities. Finally, many crisis locations suffer from lawlessness, and volunteers may be targets [17]. Volunteers may be visually and aurally different from local individuals especially with regard to clothing, footwear, and language and may therefore be specifically targeted. Robbery, injury, and various forms of kidnapping are realistic potentials that are only partly mitigated by prior self-defense training [18]. Unlike many medical education updates, personal security cannot be crafted by “just-in-time” approaches.

Volunteering to serve others is noble and laudable. It is, however, a calling fraught with risks, many of which are avoidable or mitigatable [19, 20]. Training is essential as is preparation for care outside of the highly structured and well-resourced established acute care facility. Outside of military medical service, most healthcare clinicians are principally untrained with regard to safety concerns while volunteering to render humanitarian aid—a gap that the global medical community, and medical professional organizations—should strive to repair. On-scene care is only one way to volunteer during a crisis, but it is uniquely personal and clearly demonstrates the altruism, dedication, and humanity that permeates health care regardless of where is it practiced around the world.

## Data Availability

Not applicable.
